# Early detection of cardiovascular risk markers through non-invasive ultrasound methodologies in periodontitis patients

**DOI:** 10.1515/med-2024-1003

**Published:** 2024-07-19

**Authors:** Giada Nicolosi, Martina Donzella, Alessandro Polizzi, Angela Angjelova, Simona Santonocito, Luca Zanoli, Marco Annunziata, Gaetano Isola

**Affiliations:** Department of General Surgery and Surgical-Medical Specialties, School of Dentistry, University of Catania, 95124, Catania, Italy; University Dental Clinical Center St. Pantelejmon, Faculty of Dentistry, Ss. Cyril and Methodius University in Skopje, 1000, Skopje, North Macedonia; Department of Clinical and Experimental Medicine, University of Catania, 95123, Catania, Italy; Multidisciplinary Department of Medical-Surgical and Dental Specialties, University of Campania Luigi Vanvitelli, 80138, Naples, Italy

**Keywords:** biomedicine, periodontitis, cardiovascular diseases, ultrasounds, biomarkers, inflammation

## Abstract

**Objectives:**

This narrative review aims to update the current evidence and offer insight into the new non-invasive ultrasound techniques used to early identify degenerative vascular changes in subjects with periodontitis and to investigate if these methodologies could be useful to identify subclinical cardiovascular disease (CVD) dysfunction in periodontitis patients and to monitor changes in CVD risk after periodontal treatment.

**Methods:**

Studies examining the assessment of vascular endothelial function through the latest methodologies were analyzed. Systematic reviews, observational studies, and clinical trials in the English language were identified using PubMed, Web of Science, and Google Scholar databases with key search terms such as “periodontitis,” “endothelial dysfunction (ED),” “arterial stiffness,” and “periodontal therapy.”

**Results:**

Several mechanisms are involved in the association between periodontitis and CVD. The key players are periodontal bacteria and their toxins, which can enter the circulation and infiltrate blood vessel walls. The increase in proinflammatory molecules such as interleukins and chemokines, c-reactive protein, fibrinogen, and oxidative stress also plays a decisive role. In addition, an increase in parameters of ED, arterial stiffness, and atherosclerosis, such as carotid intima-media thickness, pulse wave velocity, and flow-mediated dilatation, has been shown in periodontal patients.

**Conclusions:**

The literature today agrees on the association of periodontitis and CVD and the positive role of periodontal therapy on systemic inflammatory indices and cardiovascular outcomes. Hopefully, these non-invasive methodologies could be extended to periodontal patients to provide a comprehensive understanding of the CVD-periodontitis link from the perspective of a personalized medicine approach in periodontology.

## 1 Introduction

Periodontal disease (PD) is an inflammatory process of periodontal tissues (gingiva, bone, periodontal ligament, root cement) that results from a complex interplay between the subgingival biofilm and the host response [1,2]. In fact, the host response determines the majority of tissue damage that leads to the clinical manifestations of the disease [3]. In addition, many risk factors come into play [4,5]. These include genetic and epigenetic influences [6], lifestyle factors (such as cigarette smoking and stress) [1,7], and other systemic conditions (such as diabetes and immune deficiencies) [8,9]. They do not cause periodontitis but may predispose, accelerate, or increase disease progression [10]. On the other side, periodontitis may increase the risk for several systemic disorders, such as cardiovascular disease (CVD), diabetes, and adverse pregnancy outcomes, due to the release of bacteria and endotoxins into circulation and the increase of systemic inflammatory mediators [11,12]. These agents significantly affect endothelial cells, blood coagulation, lipid metabolism, monocytes, and macrophages through several direct or indirect mechanisms [11,13].

Recently, research has focused on highlighting the direct relationship between periodontitis and the increased risk of CVD [8,14,15]. This association may be further investigated through new ultrasonographic methodologies, such as Doppler imaging and speckle-tracking imaging (STI) [16]. Doppler imaging can be performed with different acquisition modes: continuous, pulse, and color. In general, this type of analysis provides information about hemodynamics, but only the pulse mode can be used to evaluate arterial stiffness [17]. Starting in the late 1990s, some studies presented the development of a new method, which takes the name of tissue Doppler imaging [18]. It is a color Doppler technique that provides images of tissue motion and can be applied to the walls of the carotid artery to evaluate its distensibility and, therefore, possibly find arterial stiffness [19]. STI is another semi-automated technology where tiny echo-dense speckles are tracked frame by frame. Recording their vectors, it is possible to derive secondary properties, giving information about the “deformation” of the tissue interrogated [20]. When using 2D-ST to study vascular properties, deformation patterns may be analyzed by longitudinal, radial, and circumferential directions [21,22]. The *in vivo* feasibility of ultrasound-based assessment of carotid arterial wall strain by ultrasound ST was proved recently. It proved to be a very useful method in studying the elasticity of the artery walls [23]. Many validation studies, examined and collected in a review, support its clinical use as a research tool for targeting vascular damage [16].

Currently, more consolidated non-invasive techniques are capable of assessing indices of arterial stiffness and endothelial dysfunction (ED) [24,25,26]. Among these, the most commonly performed analyses are carotid intima-media thickness (C-IMT), carotid-femoral pulse wave velocity (PWV), and brachial artery flow-mediated dilatation (FMD) [27,28]. C-IMT represents the distance between the blood-intima and media-adventitia interfaces of the carotid artery [29,30]. A B-mode ultrasound examination may show areas of increased thickening, often synonymous with atherosclerotic plaques [31,32]. PWV is based on the measurement of the propagation speed of the sphygmic wave that propagates along the arteries more or less quickly, depending on the degree of elasticity of the artery's walls [33]. It can be performed by using ultrasonographic probes or applanation tonometry, piezoelectric mechanotransducer, cuff-based oscillometry, and photodiode sensors [34,35]. FMD evaluates a phenomenon common to many blood vessels, which regulates their diameter according to the blood flow. During the execution of this method through B-mode ultrasound, it is possible to measure brachial artery diameter changes after an increase in shear stress induced by reactive hyperemia [36,37,38].

Many studies, analyzed and collected in a review, also discuss the effectiveness of periodontal and peri-implant mucositis treatment in stabilizing and normalizing clinical parameters of cardiovascular health [39,40,41]. The evidence shows that successful treatment of periodontitis has effects in the decrease of oral pathogens and inflammatory mediators released into the circulation, which is associated with a reduction of systemic inflammation and an improvement in ED [42].

Therefore, the aim of this narrative review is to analyze and update, based on the current literature, the potential advantages of the more recent ultrasound diagnostic techniques in the early identification of vascular changes in patients with periodontitis and to assess if these non-invasive methodologies are useful in the early detection of CVD and endothelial risk dysfunction in periodontitis patients at subclinical level.

## 2 Periodontitis

Periodontitis is a widely diffused chronic inflammatory-infectious disease that affects the supporting structures of the teeth. It has a high prevalence, being the sixth most common human disease [8,43,44], and the main cause of tooth loss among adult patients worldwide. Periodontitis is part of a complex group of pathologic conditions of the gingiva, bone, and periodontal ligament [1,45], called “periodontal diseases”. Generally, PD begins as gingivitis, a reversible inflammation confined to the gingiva, sustained by dental plaque [1,46,47]. The microorganisms that make up dental plaque get organized into biofilms and adhere to the tooth surface, with a progressive predominance of gram-negative bacteria, becoming resistant to host defenses and antibiotics [48,49,50,51,52,53,54,55]. Bacteria are necessary but not sufficient alone to determine the progression of the disease since the host’s specific response plays an important role [6,56,57,58]. Several agents, such as environmental factors, systemic diseases, and the host’s susceptibility, create dysbiosis [56], inducing the progressive inflammatory irreversible loss of periodontal attachment around teeth and deep periodontal tissues, with the occurrence of clinical signs of periodontitis, such as periodontal pockets, gingival recessions (REC), furcation defects, suprabony and infrabony bone defects, tooth mobility, tooth migration, and, eventually, tooth loss [57,59,60,61,62] and related low quality of life [63].

The stronger the host’s immune response will, the more extensive tissue destruction will be. People with the same amount of plaque can respond in different ways [64]. The hyperresponsive individuals produce higher concentrations of proinflammatory mediators than the hyporesponsive, inducing an aberrant reaction promoting the progression of the disease [4] through intracellular pathways involving proteins, such as the nod-like receptor family pyrin domain-containing protein-3 complex [65], playing a pivotal role in regulating the innate immune system and inflammatory signaling. Genetics and environmental factors regulate the host response: while some are adjustable (e.g., changing lifestyle habits), others are not [5,66,67]. In elderly individuals, the prevalence and severity of periodontitis are increased [68,69]. This is unclear, but the possible exposition across the years to other risk factors or the reduction of the immune function is among the hypotheses [68,70]. Environmental factors can also determine reversible epigenetic modifications [71]. Rearrangements affecting chromatin and alterations in specific genes contribute to developing and maintaining inflammatory diseases such as periodontitis [6,72]. The most known risk factors include poor oral hygiene [73], physical and psychological stress [74,75], diabetes mellitus [76], and obesity [76,77,78]. Smoking is a major factor responsible for PD susceptibility, with dose-dependent effects on the oral cavity [79]. A dose-dependent effect related to the number of cigarettes smoked per day has been demonstrated [80]. Smoking can systemically lead to an impaired immune system and local vasoconstriction, which causes a delay in the immune and inflammatory response [81]. With the same bacterial plaque, smokers compared to non-smokers have a greater number of lost teeth, deeper periodontal pockets, and greater loss of clinical attachment. Furthermore, there is a delay in diagnosing the disease due to the reduced bleeding on probing found in smokers [33,34,36].

The diagnosis of periodontitis is based on the execution of a periodontal examination, including and considering all the information collected about the patient, including intraoral photographs, etiological and predisposing factors, lifestyle habits, risk factors, level of oral hygiene, and concomitant systemic diseases. It is necessary to carry out a general inspection during which the changes in color, shape, and consistency of the gingival tissues are evaluated [82,83]. Periodontal damage assessments are a mandatory component of a comprehensive periodontal examination. Measurements made with calibrated periodontal probes are the primary way in which damage to the periodontium is assessed. These measurements include probing depths, loss of clinical attachment, and gingival REC [1,13,82]. The clinical attachment level is the distance from the amelocemental junction (CEJ) and the base of the sulcus. Gingival REC is the distance from the CEJ to the gingival margin. The gingival sulcus is a virtual space around the tooth which in physiological conditions has a depth variable between 0.5 and 3 mm and goes from the gingival margin to the base of the sulcus itself, reaching in the most apical part it reaches the CEJ [84]. Probing depth and clinical attachment loss measurements are routinely recorded at six sites around each tooth [84,85]. For each site that is probed, the presence of plaque (full mouth plaque score or FMPS) and bleeding on probing (full mouth bleeding score or FMBS) is noted. If there is gingival inflammation, the insertion of the probe will cause bleeding [1,84,86]. For the assessment of the severity and extent of the disease, the possible involvement of furcation is ascertained (using a Nabers curved probe), as well as the presence of tooth mobility [87,88]. The radiographic examination is essential for the diagnosis, intraoral radiographs of all sextants represent this to assess the damage in terms of periodontal support and bone loss caused by the disease [89]. In suprabony defects, the base of the pocket is located coronal to the alveolar crest, while infrabony defects are characterized by bone peaks at different heights and the base of the pocket located apically [90]. Periodontitis, if not adequately addressed, ultimately leads to tooth loss and contributes to systemic inflammation [1,90]. Particularly, periodontitis has been correlated with low-grade inflammation, a systemic status of chronic sub-clinical production of proinflammatory agents which represents a sneaky risk factor for many diseases such as diabetes, cardiovascular, cerebrovascular, neurodegenerative diseases, and even cancer [91].

## 3 Periodontitis and CVD

CVD represents one of the leading causes of death in the world [92]. Several conditions, such as atherosclerosis, ischemia, acute myocardial infarction, and hypertension, belong to this category [93]. Different studies have investigated the association between CVD and periodontitis over the years, and this correlation is now known [11,94,95,96]. Furthermore, these pathologies share multiple etiopathogenetic factors (e.g., genetic predisposition) and risk factors (e.g., smoking, diabetes, increasing age, and poor socioeconomic conditions) [97,98,99]. The underlying link to these two conditions appears to be low-grade and chronic systemic inflammation, in fact, the periodontal pockets present in patients with periodontitis are rich in various bacterial species, mainly gram-negative, which grow and multiply within them, inducing uninterrupted local inflammation. By proteolysis, these microbes and their toxins can destroy and invade the gingival tissues reaching the bloodstream, resulting in transient bacteremia; therefore, they may infiltrate arterial walls, contributing to inducing vascular inflammation, an earlier step for CVD [100]. Scientific evidence revealed the role of some periodontal pathogens, such as *P. gingivalis*, in the progression of atherosclerosis. In particular, it has been shown that *P. gingivalis* is able to reside in the wall of atherosclerotic vessels and epidemiological studies revealed an association between pathogen-specific IgG antibodies and atherosclerosis [101,102,103]. Interestingly, a recent study showed that *P. gingivalis* accelerates atherosclerosis via the NF-κB-BMAL1-NF-κB signaling loop [104] and though oxidation of high-density lipoprotein [105,106]. A strong association between oral health and reduced risk of coronary heart disease has been suggested. The latter seems to increase in relation to the extent of periodontitis and how many teeth are involved [107,108]. It also emerged that there is a higher risk of bacteremia in patients with large amounts of tartar and plaque after brushing teeth than in those who present less of them [109].

**Figure 1:**
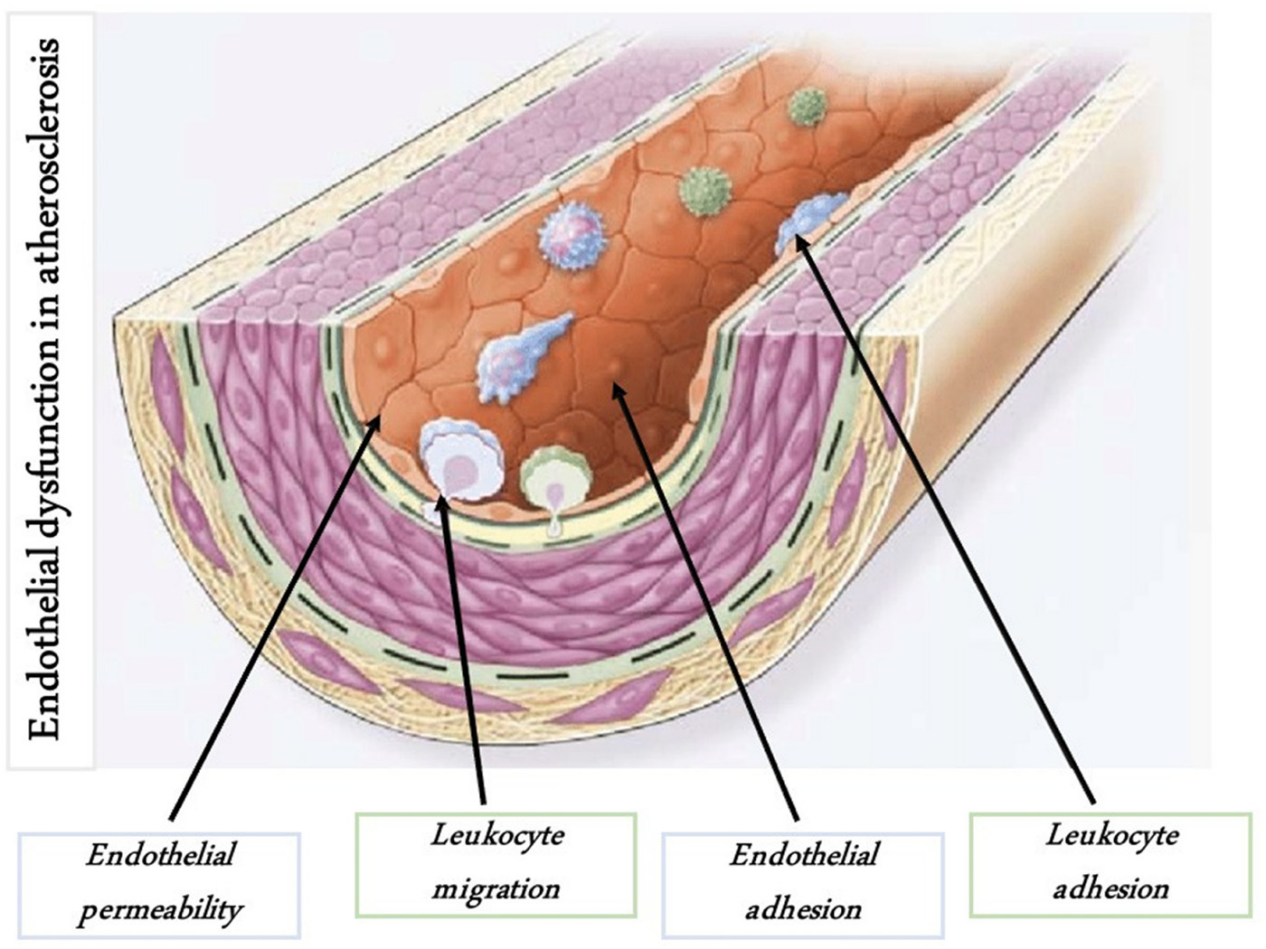
ED in atherosclerosis. First stages preceding the formation of atherosclerosis lesions occur in the endothelium. There is an increase in endothelial permeability to lipoproteins and other plasma constituents, which is mediated by NO, prostacyclin, platelet-derived growth factor, angiotensin II, and endothelin; up-regulation of leukocyte and endothelial adhesion molecules, and also migration of leukocytes into the artery wall, which is mediated by oxidized low-density lipoprotein, monocyte chemotactic protein 1, interleukin (IL)-8, platelet-derived growth factor, macrophage colony-stimulating factor, and osteopontin. From Ross [110].

The endothelium is a thin cellular monolayer that lines the inner surface of blood vessels in the cardiovascular system. The endothelial cells constitute the so-called tunica intima, which internally lines the vascular lumen, followed by the underlying tunica media, comprising smooth muscle cells (SMC), collagen, and elastic fibers [110]. Under physiological conditions, the endothelium performs its functions by modulating the vascular tone and ensuring its permeability without any obstacle to blood flow [111,112,113,114]. Endothelial cells ensure an adequate response to infections, the expression of nitric oxide (NO), the recruitment of leucocytes and cytokines, and the regulation of coagulation and fibrinolysis [100]. NO is the most important mediator of normal endothelial function, inhibiting platelet aggregation SMC proliferation, and has a significant vasodilating action. It regulates the homeostasis of the vascular system, so its reduced release, associated with the high presence of free radicals, can help determine the commonly named ED, an impairment of the normal activity of the endothelium [100,107]. Reactive oxygen species (ROS), normally produced in controlled quantities in cells, are remarkably present in periodontal tissues in patients with periodontitis. Oxidative stress and inflammation can lead to structural and functional vascular alterations and compromission of the elastic properties of the arteries, promoting the development and progression of hypertension [107,115,116]. When a significant amount of ROS is reached in progressive quantities in the oral cavity, over time there is also an increase at the systemic level [117,118]. Therefore, the main target of oxidative stress is the vascular endothelium, playing a critical role in the pathogenesis of CVD, with the resulting expression of adhesion molecules, micro-RNA, and cytokines [111,119,120,121]. Changes occurring in the endothelium, supported by the increasing inflammatory response of the host, contribute to the process of atherogenesis [110,122] (Figure 1).

Atherosclerosis is an inflammatory disease that represents the pathological basis of coronary heart disease and is among the main causes of premature death in men [123,124,125]. The peculiarity of this pathology is the formation of atherosclerotic plaques consisting of foam cells rich in ox-LDL, SMC, platelets, and other components [48,111,119,126]. As the plaque continues to grow, a thickening of the intima-media is determined, with a consequent reduction of the vessel lumen and an increase in blood pressure [127]. The total occlusion of the vessel causes thrombosis, with interruption of the blood supply and consequent ischemia; it represents an emergency. In advanced stages, the plaque can undergo rupture with partial detachment and the formation of an embolus, which can cause acute events such as ischemic heart disease and stroke [128]. Therefore, an important correlation has been identified between chronic inflammation derived from PD and oxidative stress that determines ED, promoting the development of hypertension [129]. Another adverse manifestation of structural and functional alterations of the vascular walls is arterial stiffness, which consists of a reduced capacity for arterial expansion and retraction in response to changes in blood pressure [130].

Several inflammatory mediators linked to the host response are involved in the pathogenesis and progression of CVD, but high levels of some of these can be found in patients with PD [131]. Among the most studied molecules are inflammatory cytokines, fibrinogen, and C-reactive protein (CRP) [132,133]. CRP is an inflammatory mediator whose production by the liver is stimulated by IL-6, and its purpose is to make favorable the elimination of bacteria by phagocytes; furthermore, additional hepatic production of CRP is supported in particular by endothelial cells, helping to increase local inflammation [134]. CRP also eliminates ox-LDL from the bloodstream by macrophages and in chronic inflammations. This results in the formation of foam cells, encouraging the accumulation in the vessels and contributing to the instability of the plaque due to the release of other mediators [135]. Therefore, this protein would play a key role in developing and evolving atherosclerosis and thrombosis, with greater platelet aggregation supporting thrombus formation [136]. An increase in serum levels of CRP and fibrinogen are risk factors for CVD, because they make possible the continuation of inflammation by feeding it and also act by stimulating the activation of the coagulative cascade, increasing the probability of having vasal and systemic problems [137,138]. Proinflammatory cytokines, such as IL-1, tumor necrosis factor alpha (TNF-α), and IL-6, are implicated in the pathogenesis and progression of the periodontitis and furthermore would seem to allow an increase of the atheromatous lesion inducing the formation of a greater number of spongy cells [139].

Several studies have thus demonstrated a significant association between periodontitis and CVD (Table 1).

**Table 1:** Summary of studies demonstrating a significant association between periodontitis and CVD and the mechanism behind it

Reviews	Year	Original research articles	Findings
Gurav [100]	2014	Erbesole et al. [140]	Periodontitis results in altered vascular response, and increased expression of proinflammatory cytokines and adhesion molecules, inducing vascular ED. Periodontal therapy may ameliorate the perturbed vascular endothelial function.
		Chen et al. [141]	
		D’aiuto et al. [142]	
Gibson Iii and Genco [101]	2007	Haraszthy et al. [143]	*P. gingivalis* accelerates atherosclerosis, being able to reside in the wall of atherosclerotic vessels.
		Gibson Iii et al. [144]	
Leong et al. [107]	2014	Akalιn et al. [145]	Oxidative stress in patients with PD promotes the development and progression of hypertension.
Hadi et al. [111]	2005	Akalιn et al. [145]	↑ROS in the oral cavity increases ↑ systemic level of ROS and increase ↑ adhesion molecules and cytokines in vascular endothelium and increases ↑ risk of atherogenesis and arterial stiffness.
Muñoz Aguilera et al. [129]	2020	Bragulat et al. [146]	
Cecelja and Chowienczyk [130]	2012	Lum and Roebuck [147]	
		Napoli et al. [148]	
Badimon et al. [136]	2018	Hakobyan et al. [149]	Increase in CRP and fibrinogen stimulates the activation of the coagulative cascade determining a greater risk of CVD
Munjal and Khandia [137]	2020	Bhakdi et al. [150]	
Fatkhullina et al. [139]	2016	Ohta et al. [151]	IL-1, IL-6, and TNF-α determine ↑ spongy cells ↑ risk of atheromatous lesion.
		Huber et al. [152]	
		Freigang et al. [153]	

## 4 Ultrasound vascular investigations in periodontitis patients

Today, there are several investigations that allow the identification of clinical signs intimately linked with atherosclerosis, arterial stiffness, and ED, conditions that have always been considered the basis of CVD. Among these techniques, we include Doppler imaging and STI, which originally had applications only for the study of cardiac chambers but were also approved for the study of vascular mechanisms [5,65,66]. Doppler imaging is a well-established technology based on the Doppler effect that permits the measurement of flow by the reflection of ultrasound waves. It can be performed in different modalities, including continuous, pulse, and color modes. They all measure the velocity of blood, in some cases providing useful information to identify any stenotic lesions. Pulse wave Doppler (PWD) has the advantage of being site-specific and can be used for the analysis of the assessment of arterial stiffness, for example, at the level of the carotid artery [19,154]

These methods and those that we will discuss in the following paragraphs are used to assess cardiovascular risk in patients with predisposing conditions, including the burden of periodontitis.

### 4.1 STI

STI is a 2D analysis of the spatial dislocation of speckles, defined as spots or marks, generated by the interference between analyzed structures and ultrasound waves [20]. Speckles are tracked consecutively from frame to frame to obtain information about arterial mechanics, including displacement, velocity, strain (*ε*), and strain rate (SR). A review by Teixeira et al. [16] explains that the displacement may be analyzed in longitudinal, radial, and circumferential directions, even if this technique works better on measurements in the same direction as the ultrasound probe [155]. Nevertheless, circumferential analysis is the one typically performed, including strain and SR determinations. This is a useful technique in the evaluation of new elastic properties of vascular walls. Circumferential vascular mechanics may thus serve as a surrogate of local vascular stiffening, having a significant association with PWV [16,156]. The validity of results depends on image quality and on the accuracy of tracking, which must be performed by an experienced operator [157].

### 4.2 CIMT

For the first time, a link between the severity of periodontitis and intima-media thickness of the carotid artery (CIMT) was found by Beck et al. in a randomized clinical trial [158]. A few years later, the same conclusions were drawn from other studies [159]. In 2008, a case–control study by Cairo et al. confirmed the existence of this association not only in the elderly population but also in young, systemically healthy individuals [160]. The carotid IMT can be measured simply, noninvasively, and reproducibly through B-mode carotid ultrasound. B-mode indicates an ultrasound execution mode in which the image is constructed by converting the reflected waves into signals whose brightness is proportional to the intensity of the echo and expressed with a scale of shades of grey [161]. Even today, most ultrasound modalities, including the B-mode, provide two-dimensional images. Two bright lines exist in a 2D grey-scale image of the carotid artery. The upper one is the interface between the blood and intima layer and the lower one is the interface between the media layer and the adventitia layer. Cor CIMT defines the distance between these two bright lines [162,163].

**Figure 2:**
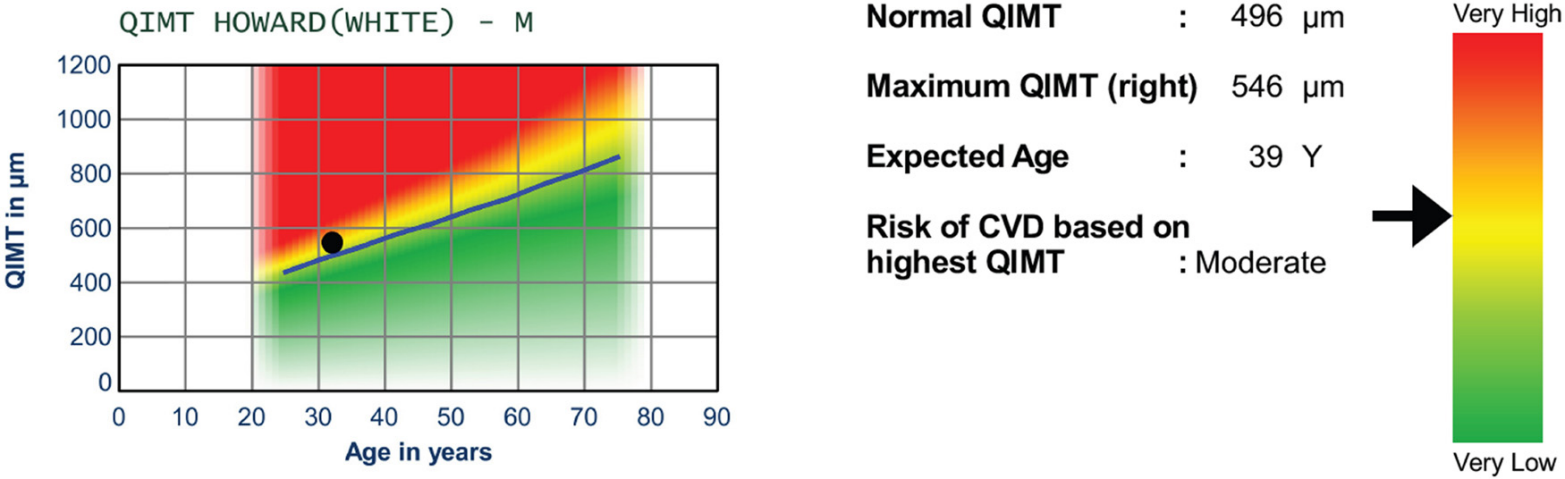
Evaluation of cardiovascular risk assessment according to CIMT maximum values. In this case, the curve indicates a moderate risk for cardiovascular events.

The carotid artery is classified into three segments, each approximately 1 cm in length. The most proximal segment, the common carotid (CCA) represents the 1-cm straight segment, which is located before the bifurcation into its internal and external branches, the internal carotid artery and external carotid artery. The CCA far wall is the easiest segment to be examined and the most commonly used in clinical studies [164]. There are many recommendations on CIMT acquisition and measurement that are published and updated periodically. These concern the position of the patient during the examination, the type of probe, and all the technical parameters to be set to obtain an excellent image quality [165]. CIMT values of around 0.5 mm are considered normal in young adults; instead, CIMT above 1.0 mm is regarded as abnormal and if the IMT is above 1.2 mm, the patient is considered to be at high risk of CVD [31,166] (Figure 2). Many clinical trials found that mean IMT was higher in patients with PD than in patients without it [154,158,167–169].

### 4.3 PWV

**Figure 3:**
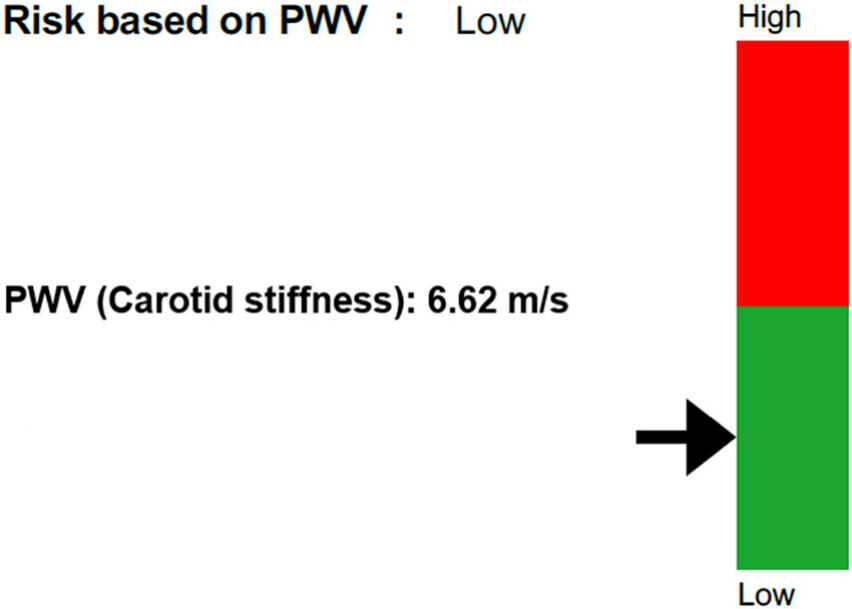
Evaluation of cardiovascular risk assessment according to PWV values. In this case, PWV assessment indicates a low risk for cardiovascular events.

Currently, PWV is the gold standard method to assess arterial stiffness [170,171] (Figure 3). It is a non-invasive measurement of the distance between two recording sites, covered by pressure waves on the transit time [172,173]. PWV = (distance/transit time). PWV can be measured from different sites of the vascular system, but the measurement is preferably taken in the carotid-femoral pathway [174,175]. Also, the brachial-ankle PWV (baPWV) is considered valid, especially as a marker of the stiffness of the large to medium-sized arteries [176]. PWV is not the same as PWD since PWD measures the speed of the pulse at one specific point, whereas the PWV test measures the speed of propagation of one single wave, i.e. how long does it take for the pulse wave (with an imaged maximum speed of blood flow of 80 cm/s at a specific point – this is PWD) to travel from A (the Heart) to (B). Currently, many devices are validated to perform PWV. Some use applanation tonometry, piezoelectric mechanotransducers, cuff-based oscillometry, or photodiode sensors [177]. The ultrasound-based technique is probably the most accurate but often remains confined to clinical research, like MRI [34]. There are several factors that can affect the accuracy of the results [178]. Indeed, it depends on whether the carotid-femoral pathway measurement can be obtained directly or not [179]. A carotid-femoral PWV value greater than 10 m/s is considered to be an index of large artery stiffening. Threshold values (such as >90th percentile or >75th percentile of normal values for PWV dependent upon age) may be more accurate reference values for identifying people at increased CV risk [180,181]. To date, only a few studies have evaluated the association between PWV and periodontitis in otherwise healthy subjects. Among these clinical trials, not all came to the same conclusions. Some showed significantly higher values of PWV in a patient with periodontitis [182–184], while others did not [185].

### 4.4 FMD

FMD represents a typical phenomenon of blood vessels that can dilate in response to physical and chemical changes. It is a non-invasive method based on the measurement of brachial artery diameter changes after an increase in flow, precisely shear stress [186]. The brachial artery diameter is measured before inflation (to 200–300 mmHg) and after release (5 min later) of a sphygmomanometer cuff placed on the forearm. Despite its apparent simplicity, its application is technically challenging and requires a rigorous protocol and a trained operator. For these reasons, FMD has only recently reached an adequate methodological standardization level to be proposed as a surrogate endpoint in clinical trials [187,188]. To acquire images with a good resolution, it is necessary to comply with a series of technical precautions explained in the guidelines. FMD results are typically expressed as the percentage increase in the artery diameter above baseline. Lower FMD values than normal are synonymous with ED [36].

In 2003, in a clinical trial, Amar et al. [189], for the first time, demonstrated the association between PD and brachial artery ED. In fact, periodontal subjects who participated in the study were found to have significantly impaired FMD values. Many other case–control studies like the previous one, demonstrated the same [190–192].

### 4.5 Alterations in ED parameters in periodontal patients compared with healthy patients

Current evidence confirms the association between periodontal inflammation and increased cardiovascular risk shown by impaired vascular health in subjects with chronic periodontitis (Table 2). Periodontitis may be an insidious cause of ED, arterial stiffness, and atherosclerosis, as evidenced by the increase in specific cardiovascular parameters. Specifically, a comparison between healthy and periodontal subjects revealed significant differences in FMD, CIMT, and PWV values (Table 3). FMD values, expressed as a percentage, were significantly lower in subjects with PD, found to be impaired from the reference values and placed in the intermediate cardiovascular risk range. CIMT values, expressed in millimeters, were higher in periodontal patients and very close to the critical threshold of abnormality. Finally, PWV values, expressed in m/s, were also higher in the periodontal group and considered an index of early-stage arterial stiffness. However, current evidence is limited by the lack of randomized controlled trials. In this sense, the current data should be interpreted only in terms of correlation rather than causality with regard to the impact of periodontitis on cardiovascular parameters.

**Table 2:** Summary of evidence from the past twenty years regarding increased parameters related to ED, arterial stiffness, and atherosclerosis in healthy patients and in periodontal patients

**Study**	**Females (F) %**	**Type of study**	**Analyzed parameter**	**Healthy patients (H)**	**Periodontal patients (P)**	***p*** **-value**
Amar et al. [189]	HF: 38%	Cross-sectional	FMD	11.7 ± 5.3%	7.8 ± 4.6%	*p* = 0.005
	PF: 39%					
Mercanoglu et al. [190]	HF: 42%	Cross-sectional	FMD	19.4 ± 8.1%	8.4 ± 4.0%	*p* < 0.001
	PF: 25%					
Seinost et al. [192]	HF: 51%	Cross-sectional	FMD	8.5 ± 3.4%	6.1 ± 4.4%	*p* = 0.002
	PF: 63%					
Blum et al. [191]	PF: 55%	Cross-sectional	FMD	16.60 ± 7.86%	4.12 ± 3.96%	*p* = 0.001
Söder et al. [159]	HF: 55%	Cross-sectional	C-IMT	0.58 ± 0.09 mm	0.66 ± 0.12 mm	*p* < 0.01
	PF: 50%					
Cairo et al. [160]	HF: 47%	Cross-sectional	C-IMT	0.72 ± 0.07 mm	0.82 ± 0.13 mm	*p* < 0.001
	PF: 47%					
Jockel-Schneider et al. [184]	NA	Cross-sectional	PWV	7.7 ± 1.9 m/s	9.1 ± 2.2 m/s	*p* = 0.001
Houcken et al. [182]	HF: 45%	Cross-sectional	PWV	7.36 m/s	8.01 m/s	*p* = 0.029
	PF: 44%					

F: females; H: healthy; P: periodontal; NA: not available.

**Table 3:** Summary of relevant studies evaluating the efficacy of periodontal therapy

Authors	Females (F) %	Type of study	Type of exam	Baseline	After periodontal treatment	*p*-value (baseline vs after therapy)
Piconi et al. [209]	HF: 38%	Cross-sectional	CIMT	0.49 ± 0.02 mm	0.37 ± 0.03 mm	*p* < 0.001
	PF: 39%					
Kapellas et al. [210]	HF: 40%	Randomized Clinical Trial	CIMT	0.64 ± 0.14 mm	0.63 ± 0.14 mm	*p* = 0.13
	PF: 44%					
Vidal et al. [212]	PF: 62%	Prospective study	PWV	13.7 ± 2.4 m/s	12.5 ± 1.9 m/s	*p* < 0.01
Kapellas et al. [210]	HF: 40%	Randomized Clinical Trial	PWV	8.27 ± 1.30 m/s	8.44 ± 0.92 m/s	*p* = 0.06
	PF: 44%					
Houcken et al. [182]	HF: 45%	Cross-sectional	PWV	8.00 ± 1.8 m/s	7.82 ± 1.6 m/s	*p* = 0.13
	PF: 44%					
Jockel-Schneider et al. [214]	NA	Randomized Clinical Trial	PWV	8.92 ± 2.23 m/s	8.85 ± 2.27 m/s	*p* = 0.78
Mercanoglu et al. [190]	HF: 42%	Prospective study	FMD	8.4% ± 4.0%	17.7% ± 5.7%	*p* < 0.001
	PF: 25%					
Seinost et al. [192]	HF: 51%	Prospective study	FMD	6.1% ± 4.4%	9.8% ± 5.7%	*p* = 0.003
	PF: 63%					
Blum et al. [191]	PF: 55%	Prospective study	FMD	4.12% ± 3.96%	11.12% ± 7.22%	*p* = 0.007

The table shows carotid intima-media thickness (CIMT), pulse wave velocity (PWV), and flow mediated dilatation (FMD) values at baseline and after periodontal therapy.

Treating periodontitis can improve endothelial function and be an important preventive tool for CVD.

## 5 Periodontal therapy as CVD risk reduction

Periodontal therapy aims to remove the bacterial biofilm from the supra and subgingival dental surfaces. This is a non-surgical approach that involves the instrumentation of these surfaces, performed with hand (i.e., curettes) or power-driven (i.e., sonic/ultrasonic devices) instruments [193–195]. In addition to the instrumentation, various physical or chemical agents can be used to help reduce the microbial load [196,197]. The non-surgical approach also includes recommendations given to patients on lifestyle changes and on risk factors control [198]. As previously mentioned, periodontitis includes, among its risk factors, smoking and diabetes, which are also common to CVDs. The literature on the subject has evidence that smoking cessation and glycemic control have positive results both on the periodontal condition of patients and on the reduction of cardiovascular risk [76,199–201]. Other studies prove that periodontal therapy can help patients with glycemic control.

According to recent evidence, subgingival instrumentation demonstrated a mean reduction of PPD of 1.7 mm at 6/8 months, a mean proportion of closed pockets of 74%, and a mean reduction of bleeding on probing (BOP) of 63%. Deeper sites (>6 mm) resulted in a greater mean PPD reduction of 2.6 mm [202]. The stability of these parameters testifies to a decrease in inflammation of the periodontal tissues; for this reason, periodontal therapy has also been widely demonstrated to reduce various cardiovascular risk factors positively. Particularly, results have been reported in improving serum inflammation biomarkers (CRP, Ils), clinical indices of arterial stiffness, atherosclerosis, and endothelial function [203]. A lot of studies evaluated serum levels of CRP before and after periodontal therapy. From the majority of these, it seems that periodontal therapy has a positive role, but it is also true that actually, the evidence includes both randomized and non-randomized trials and both short- and long-term studies [204–207]. Increased risk for the future formation of atherosclerosis is estimable by performing CIMT. The association between periodontitis and an increase in carotid thickness has already been demonstrated previously [208]. Other authors concluded that also periodontal therapy could influence this vascular parameter, obviously in a positive way. Piconi et al. showed reduction of IMT at the carotid bifurcation, at 1 cm from the bifurcation and at 2 cm from the bifurcation, 6 and 12 months after initiation of PD [209]. Kapellas et al. reported similar conclusions 12 months after [210]. Desvarieux et al. detected a difference in CIMT among participants of approximately 0.1 mm between the baseline and after a 3-year follow-up [211].

Regarding arterial stiffness, as already said, the reference method of diagnosis is PWV. Currently, evidence still has few studies evaluating the changes in PWV values before and after periodontal therapy. Vidal et al. have achieved results, demonstrating an improvement 6 months after periodontal therapy in hypertensive patients [212]. Ren et al. evaluated baPWV, revealing a remarkable decrease in it, with a mean difference of −0.58 m/s between the baseline and 1 month after periodontal treatment [213]. Conversely, Kapellas et al. [210] in a randomized controlled trial did not reach significant results, with a 12-month follow-up. So it also emerged from the article by Houcken et al. [182] which considered a follow-up of 6 months. Similarly, in a randomized controlled trial, Jockel-Schneider et al. did not show a relevant difference in PWV values 12 months after periodontal therapy [214]. The difference in the results obtained probably depends on the different selection criteria adopted by each clinical study. Furthermore, it has been asked whether the treatment of periodontitis can improve endothelial function. The answer has come from many studies conducted over the past 15 years. In 2004, Mercanoglu et al. noticed a correlation between changes in periodontal clinical parameters and changes in FMD values, after periodontal therapy [190]. Seinost et al. found similar results, valuating FMD values at baseline and 3 months after PD [192]. In 2007, in a randomized clinical trial, Tonetti et al. concluded that 6 months after intensive periodontal therapy, individuals had a 2.0% rise of FMD [215]. In the same year, Blum et al. reported a significant improvement in endothelial function in periodontal patients 6 months after therapy [191]. Many mechanisms can explain these effects. Surely, periodontal therapy has several benefic effects on endothelial function; one of the most important is the increase of NO bioavailability [216]. The three different types of measurements reported are mostly in agreement in associating periodontitis with an increase in the parameters that indicate cardiovascular risk conditions. Moreover, the majority also believe that periodontal treatment has beneficial effects in reducing these parameters. Speckle tracking analysis, precisely due to the fact that it is a recent evaluation, not well standardized, has not yet been used in periodontal patients to evaluate the parameters before and after periodontal therapy. At the moment, there is no evidence yet, but it is hoped that this analysis will become an alternative method for the detection of vascular parameters also in periodontal patients.

A summary of relevant studies that evaluated the efficacy of periodontal therapy is given in Table 3.

As reported in Table 3, although all authors agreed in attributing a beneficial effect of periodontal therapy on patients’ cardiovascular health, when comparing values before and after periodontal treatment, a significant change was found in just over half of the studies analyzed.

Since long ago, many authors have also concentrated on researching the effects of periodontal therapy on CVD. Lastly, some studies considered the impact of periodontal therapy on a transient systemic perturbation, similar to what happens next to other invasive dental procedures [217]. Graziani et al. compared two periodontal therapy approaches, full-mouth instrumentation (FM-SRP) and quadrant scaling and root planning (Q-SRP). In FM-SRP, the patients are treated in two sessions held within 24 h; instead, Q-SRP provides an instrumentation session for each quadrant, each one at a distance of one week [218–220]. This comparison revealed that only FM-SRP is responsible for a transient condition of systemic inflammation, witnessed by the increase of inflammatory markers like TNF-a, IL-6, and CRP. Therefore, it is recommended not to use this approach in patients who already have high-risk conditions [221].

Table 4 resumes the principles of the main ultrasound investigation methods in periodontitis patients and the advantages and disadvantages in relation to the disease.

**Table 4:** Summary of the main ultrasound investigation methods and their relation with periodontitis

Ultrasound vascular investigation	Principles	Relation with periodontitis	Advantages	Disadvantages	References
STI	Utilizes echocardiographic grayscale images to track speckles or natural acoustic markers in the myocardium, providing a detailed analysis of myocardial strain and deformation.	Emerging evidence suggests that periodontitis may contribute to subclinical cardiovascular changes detectable by STI, although more research is needed to establish a direct link.	Non-invasive	Operator-dependent	[222,223]
			Provides detailed myocardial strain analysis	Requires high-quality images	
			Detects subtle myocardial dysfunction	Limited by acoustic window	
CIMT	Measures the thickness of the carotid artery's intima and media layers using B-mode ultrasound, indicating early atherosclerotic changes​	Strongly correlated with periodontitis; chronic inflammation from periodontitis can lead to increased CIMT, indicating a higher risk of atherosclerosis	Non-invasive	Limited to larger arteries	[162,209,210]
			Simple and widely available	Does not directly measure plaque stability	
			Predictive of cardiovascular events	Operator-dependent	
PWV	Assesses arterial stiffness by measuring the speed of the pressure wave traveling between two arterial sites, often the carotid and femoral arteries	Increased PWV is observed in patients with periodontitis due to systemic inflammation leading to arterial stiffness	Non-invasive	Requires specialized equipment	[170,212,214]
			Measures arterial stiffness	Sensitive to blood pressure changes	
			Provides prognostic information on cardiovascular risk	May be influenced by other cardiovascular conditions	
FMD	Evaluates endothelial function by measuring the dilatory response of an artery (typically the brachial artery) to increased blood flow induced by temporary occlusion (usually using a blood pressure cuff)​	FMD is impaired in periodontitis patients. This reflects ED, which is a key early event in atherosclerosis development	Non-invasive	Operator-dependent	[187,190,192]
			Assesses endothelial function	Requires strict standardization	
			Can detect early vascular changes	Affected by various physiological factors	

## 6 Conclusions and future prospects

This review supports the utility of many non-invasive ultrasound techniques as a tool for the early diagnosis of CVD by identifying signs of atherosclerosis, arterial stiffness, and ED.

Despite these interesting results, it is necessary to specify that the evidence for differences between healthy and periodontitis subjects in cardiovascular ultrasound parameters is actually limited. In this regard, the current evidence should be understood in simple terms of correlation since many of the articles analyzed are observational case–control studies.

Unanimous evidence is still lacking in adopting these non-invasive techniques in the diagnosis of subclinical pictures of CVD in periodontal patients. These techniques in clinical practice are commonly used for primary and secondary prevention in all those patients with cardiovascular risk factors but are still rarely used in periodontal patients to identify early stages of cardiovascular changes. Hopefully, these screening tools can be extended to all periodontal patients in the future, possibly by using specific software that allows making a three-dimensional analysis of the vascular structures to overcome the limitations of two-dimensional exams. With new tools for a comprehensive understanding of the CVD-periodontitis link, it will be possible to refine diagnostic and therapeutic goals and to tailor them to each patient, in the perspective of a personalized medicine approach also in the field of periodontology, extending the benefit of periodontal treatment not only to oral health but also to systemic health, through the prevention and treatment of the systemic inflammation underlying CVD.
